# Exome Sequencing Identifies a Novel Gene, *WNK1*, for Susceptibility to Pelvic Organ Prolapse (POP)

**DOI:** 10.1371/journal.pone.0119482

**Published:** 2015-03-04

**Authors:** Shuquan Rao, Jinghe Lang, Lan Zhu, Juan Chen

**Affiliations:** 1 National Laboratory of Medical Molecular Biology, Institute of Basic Medical Sciences, Chinese Academy of Medical Sciences & Peking Union Medical College, Tsinghua University, Beijing, 100005, PR China; 2 Department of Obstetrics and Gynecology, Peking Union Medical College Hospital, Chinese Academy of Medical Sciences & Peking Union Medical College, Beijing, 100005, PR China; Kunming Institute of Zoology, Chinese Academy of Sciences, CHINA

## Abstract

Pelvic organ prolapse (POP) is a common gynecological disorder; however, the genetic components remain largely unidentified. Exome sequencing has been widely used to identify pathogenic gene mutations of several diseases because of its high chromosomal coverage and accuracy. In this study, we performed whole exome sequencing (WES), for the first time, on 8 peripheral blood DNA samples from representative POP cases. After filtering the sequencing data from the dbSNP database (build 138) and the 1000 Genomes Project, 2 missense variants in *WNK1*, c.2668G > A (p.G890R) and c.6761C> T (p.P2254L), were identified and further validated via Sanger sequencing. In validation stage, the c.2668G > A (p.G890R) variant and 8 additional variants were detected in 11 out of 161 POP patients. All these variants were absent in 231 healthy controls. Functional experiments showed that fibroblasts from the utero-sacral ligaments of POP with *WNK1* mutations exhibited loose and irregular alignment compared with fibroblasts from healthy controls. In sum, our study identified a novel gene, *WNK1*, for POP susceptibility, expanded the causal mutation spectrums of POP, and provided evidence for the genetic diagnosis and medical management of POP in the future.

## Introduction

Pelvic organ prolapse (POP) is a global health problem that affects approximately 50% of women over 50 years of age [1,2]. Although POP is not life-threatening, it often causes an adverse impact on daily activities and quality of life because of pelvic discomfort and urinary dysfunction [3,4]. Moreover, one in five women affected with POP will undergo surgical repairs in her lifetime, and an estimated 30% of women require re-operation, which represents an enormous financial burden on society [5].

Although multiple risk factors, including advancing age, vaginal childbirth, decompensation and obesity, have been identified to increase the risk of POP [3,6,7], the underlying mechanisms are poorly understood. One potential POP mechanism is biomechanical weakness of the pelvic support tissues, which has been attributed to a disturbance in connective tissue metabolism [8]. The connective tissue contains relatively few cell types; the majority of cells are fibroblasts, which produce fibrillar components, namely, collagen and elastin, to form the extracellular matrix (ECM). The fibrillar components facilitate cell attachment and alignment, which provides the main source of biological forces to maintain the correct position of the uterus. In POP patients, the collagen content is reduced, which could lead to irregular fibroblast cell alignment and subsequently decreased contractibility [9,10].

Epidemiologic studies have shown that if parents have suffered from POP, the relative risk for their children to develop POP is two- to three-fold [11], and the risk of POP is 5-fold higher in the siblings of women with advanced POP [12]; these findings provide evidence that POP may be hereditary. To date, a number of susceptibility genes have been identified through association studies [13–15] and linkage analysis [16]. However, all identified variants were common, and the rare variants that could play more important roles in the etiology of POP remain to be identified.

In addition, because of limitations in research strategies, researchers have screened for POP susceptibility genes in only a narrow range, which has undoubtedly missed many potential susceptibility genes. Until now, most genes identified were fibroblasts or ECM-associated genes, such as MMP, COL3A1 and LAMC1 [14,16,17].

Exome sequencing has high exonic coverage and accuracy, and it has been successfully used to identify pathogenic gene mutations in a number of diseases. In this study, we performed whole exome sequencing (WES) in eight sporadic POP patients with highly homogeneous symptoms, for the first time, to screen for susceptibility to rare POP variants.

## Materials and Methods

### Subjects and blood samples

This study was approved by the Peking Union Medical College Hospital Ethics Committee (project No. S-450). Three hundred ninety-two participants, which included 161 patients with POP (63.7 ± 12.4 years old) and 231 healthy controls (62.2 ± 10.8 years old), were recruited from the Beijing Union Medical College Hospital. Family history of each participant was investigated during the outpatient procedure and all of them were from unrelated families. All participants provided written informed consent. All participants were clinically examined by at least two senior gynecologists using the criteria of the International Continence Society to determine the stage of POP (pelvic organ prolapse quantification, POP-Q) [18]. Patients with stage II POP or lower were excluded. Both premenopausal and postmenopausal POP patients were recruited. All controls were healthy women, postmenopausal for at least two years, had no use of hormone therapy in the previous year and no prior history of prolapse surgery. For both groups, individuals with chronic pelvic inflammatory diseases, endometriosis, gynecological malignancies or connective tissue diseases were excluded.

All subjects were of Chinese Han origin and were geographically located in northern China. Ethylenediaminetetraacetic acid—anti-coagulated venous blood samples were collected, and genomic DNA was extracted from lymphocytes using the FlexiGene DNA kit (QIAGEN, USA) according to the manufacturer’s instructions.

### Exome capture and next-generation sequencing

Exome capture was performed using the Roche NimbleGen SeqCap EZ Human Exome Library v3.0 kit (Roche, UK) according to the manufacturer’s standard procedures. Paired-end sequencing was conducted on an Illumina Hiseq 2000 platform (Illumina, USA) with a read length of 100 bp using standard protocols previously described [19]. For each DNA sample, we obtained more than 10 Gb of clean sequence data with more than 50 x read depth. The raw image was processed for base calling using Illumina Pipeline Software.

### Read mapping, variant calling and annotation

Sequence reads in each individual were aligned to the human reference genome (NCBI build GRH37) using the Burrows-Wheeler Aligner (BWA) [20]. The genome analysis toolkit (GATK) [21], SAMtools [22] and Picard tools were used to remove duplicates and “false” mutations introduced by library construction and to recalibrate map quality scores. Single nucleotide variants (SNVs) were identified by the Unified Genotyper module in GATK, and insertion-deletions (indels) were detected with a GATK Indel Genotyper V2. All results followed the standard filtering criteria. Only single nucleotide polymorphisms (SNPs) with a read coverage ≥ 4 x, a Phred-scaled SNP quality ≥ 20, and a distance between two adjacent SNPs no less than 5 bp were retained. All variants were annotated using ANNOVAR [23].

### PCR and Sanger sequencing

Validation for 4 varaints identified in *WNK1* and scanning of the entire coding regions of *WNK1* were performed using the standard Sanger sequencing method. Primer pairs surrounding the variants (S1 Table) and covering the entire coding regions, of *WNK1* (S2 Table) were designed with Primer Premier 5.

A polymerase chain reaction (PCR) reaction was performed using a 2 x PCR master mix (TIANGEN, China), 40 ng of genomic DNA and 5 pmol each of forward and reverse primers.

The cycling conditions involved an initial step at 95°C for 5 min, followed by 35 cycles of denaturation at 95°C for 30 s, annealing at 45–60°C for 30 s and extension at 72°C for 45 s. PCR products were resolved on 1% agarose gels, stained with ethidium bromide (1 μg/ml), visualized with the Gene Genius Bio-imaging system (Syngene, UK), and then sequenced in TsingKe (China).

### Establishment of primary fibroblast cultures from the uterosacral ligament

Cultures were established from the uterosacral ligament within 6 h of post-surgical excision as previously described [24]. Briefly, biopsies were washed 3 times in 1× PBS and incubated in 0.5 mg/ml collagenase I (Roche, UK) for 2 h in a 37°C/5% CO_2_ humidified atmosphere. Following centrifugation, the cells were pelleted and re-suspended in M199 medium, which was supplemented with 15% FBS (Gibco, USA), 100 units/ml penicillin and 100 μg/ml streptomycin (Gibco, USA), 1% non-essential amino acids (Sigma-Aldrich, UK) and 250 μg/ml amphotericin-B (Sigma-Aldrich, UK), at 37°C in an atmosphere of 5% CO_2_ for 3 h. Non-adherent cells were collected by centrifugation, adjusted to a suitable concentration of 150,000 cells/ml, and cultured for experiments.

### Immunohistochemistry (IHC)

IHC was performed using standard methods. Fibroblasts were fixed in 4% paraformaldehyde (PFA) for 15 min at room temperature (RT), penetrated by 0.5% Triton X-100 for 7 min, and then blocked in 3% BSA for 1 h at RT. After incubation with primary antibody at 4°C overnight, the cells were treated with polymer helper and poly peroxidase-anti-Rabbit IgG (ZSGB, China) for 10 min each and subsequently incubated in DAB complex (ZSGB, China) for visualization. The nuclei were stained with hematoxylin (ZSGB, China). The primary antibodies used included mouse anti-Cytokeratin 19 (1:100, ZSGB, China) and mouse anti-Vimentin (1:150, ZSGB, China).

### Statistical analysis

The programs SPSS and Microsoft Office Excel 2007 were used for data analysis. *P* < 0.05 was considered to be significant in all experiments.

## Results

### Clinical features of POP individuals

We performed exome sequencing in 8 patients with a clinical diagnosis of POP. Their lab IDs were P28, P51, P129, P136, P140, P142, P151 and P153. Because environmental factors and medical history could greatly increase a woman’s risk of suffering from POP, we selected POP patients for exome sequencing strictly according to the following criteria: 1) premenopausal (as young as possible; the youngest patient was 30 years old); 2) no stress urinary incontinence (a disease with causes similar to POP); 3) no medical history of chronic pelvic inflammatory disease, endometriosis, gynecological malignancies, chronic obstructive pulmonary disease (COPD) or other chronic respiratory diseases, connective tissue disorders or pelvic surgery; and 4) no hormones within the previous year. None of the patients belonged to extended pedigrees.

### Exome sequencing identified a susceptibility gene, *WNK1*

On average, exome sequencing generated 10.9 Gb of sequence data per individual as paired-end, 2 x 100 bp reads. After quality control, exon regions of 60 Mb were targeted with a mean coverage of 111 folds, and at least 97% of nucleotides were sequenced at least four times (Table 1). These datasets ensured the capture of coding mutations with considerable sensitivity and specificity. On average, 189,194 SNPs and 14,368 indels were identified per subject.

**Table 1:** Overview of exome sequencing data.

Exome Capture Statistics	P28	P51	P129	P136	P140	P142	P151	P153
Target region (bp)	64190747	64190747	64190747	64190747	64190747	64190747	64190747	64190747
Raw reads (Gb)	13.7	9.4	12.1	10.4	10.4	12.9	8.7	9.3
Single read length (bp)	100	100	101	101	100	100	100	100
Raw data yield (Mb)	14735	10131	13045	11152	11137	13849	9328	9996
Data mapped to target region (Mb)	8977	6390	7457	6369	7033	8295	6679	6122
Mean depth of target region (X)	139.8	99.5	116.2	99.2	109.6	129.2	104.1	95.4
Coverage of target region (%)	99	98.7	98.6	98.4	98.9	98.9	98.6	98.7
Fraction of target covered > = 4X (%)	97.9	97.5	97.3	97	97.8	97.8	97.6	97.5
Fraction of target covered > = 10X (%)	96.8	96.2	96.1	95.7	96.6	96.7	96.4	96.2
Fraction of target covered > = 20X (%)	95.7	94.3	94.7	93.8	94.9	95.6	94.2	94.5
Data mapped to X (bp)	805127070	542746799	655988836	565652939	581250962	757472197	573572126	530501987
Data mapped to Y (bp)	7029026	3671239	5220464	4575333	3924501	6828294	3431305	3615369

**Fig 1:**
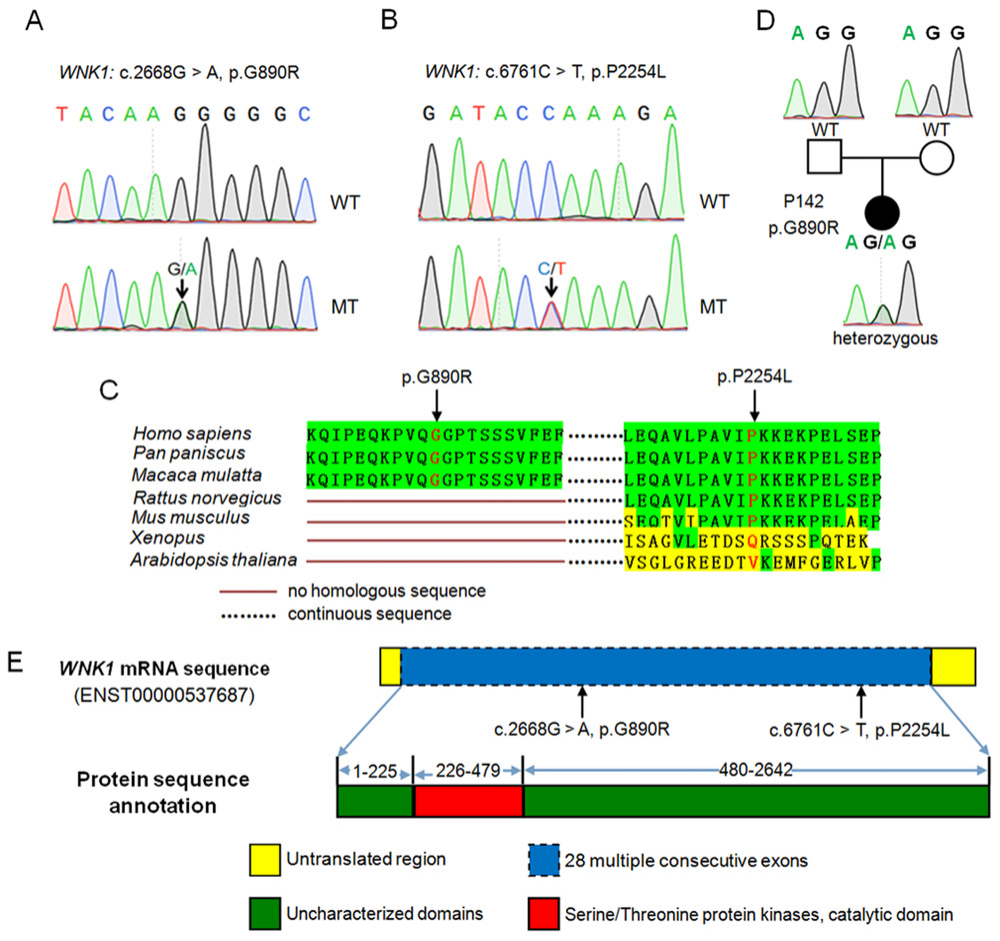
Exome sequencing identified two missense variants of *WNK1* in POP patients. (A and B) Sanger sequencing chromatograms of the two *WNK1* mutations. The positions of the mutations are indicated by an arrow. (C) Comparative protein alignment of WNK1 protein in *Homo sapiens*, *Pan paniscus*, *Macaca mulatta*, *Rattus norvegicus*, *Mus musculus*, *Xenopus* and *Arabidopsis thaliana*. The mutated amino acids were shown in red. (D) DNA sequence chromatograms showing de novo heterozygous mutation of c.2668G > A (p.G890R) in P142. (E) Schematic representation of the human *WNK1* gene (top) and protein (bottom). WNK1 contains 2,642 amino acids, serine/threonine protein kinases catalytic domain included. The mutated amino acids (*) are highlighted in red.

We first removed the common SNPs recorded in dbSNP 138 and the 1000 Genomes Project database and then excluded synonymous and intronic variants outside of splice junctions, which were unlikely to be causative. Because variants in untranslated regions (UTRs) were less likely to impair gene functions, we focused on variants that resided in coding sequences (CDS) and splice-acceptor and-donor sites. Since collecting family information of POP suggested that autosomal dominant transmission was the most likely mode of transmission for POP [16], therefore we selected candidate genes according to the following criteria. 1) Genes had either no fewer than two variants or one variant that occurred twice or more. 2) For any candidate gene, each case had no more than one variant, which was in accordance with dominant inheritance. 3) Variants of one candidate gene in any case were singe heterozygous. A total of 10 genes met the criteria mentioned above (S3 Table). To find the really causative variant of POP, we applied Sorting Intolerant From Tolerant (SIFT) and Polymorphism Phenotyping Version 2 (PolyPhen-2) software to predict the possible effects of variants on protein functions (S3 Table). Finally, *WNK1* was selected for the following reasons. 1) Up to 4 variants, namely c.4T> A (p.S2T), c.227A> G (p.E76G), c.2668G > A (p.G890R) and c.6761C> T (p.P2254L) were detected in six POP patients (Table 2). 2) All the four variants were predicted to affect the structures or functions of *WNK1* either by SIFT or PolyPhen-2 software. 3) WNK kinases were reported to positively regulate canonical Wnt/b-catenin signaling [25], repression of which could lead to POP [26,27]. Two variants, c.2668G > A (p.G890R) and c.6761C> T (p.P2254L), were validated through bidirectional Sanger sequencing (Fig. 1A and 1B). Alignment of orthologous WNK1 in seven species, including *Homo sapiens*, *Pan paniscus*, *Macaca mulatta*, *Rattus norvegicus*, *Mus musculus*, *Xenopus* and *Arabidopsis thaliana*, showed that p.G890R and p.P2254L in WNK1, are highly conserved (Fig. 1C). We further examined whether the two mutations were transmitted from their parents or not. Since POP patients were all very old, it was difficult to get DNA samples from both parents. Only c.2668G > A (p.G890R) in P142 was analyzed at last. Sanger sequencing results showed that c.2668G > A (p.G890R) occurred only in affected offspring, suggesting that this mutation was *de novo* (Fig. 1D).

**Table 2:** Brief information regarding the variants that occurred in at least two patients after filtering.

Gene Symbol	Transcription ID	Individuals sharing the variant	Mutation type	Chr. (position)^1^	Nucleotide change	Protein level change	SIFT prediction	PolyPhen-2 prediction	Gene function^2^
WNK1	ENST00000537687	P153	SNV	Chr12: 862735	c.4T>A	p.S2T	Damaging	Probably damaging	Members of the WNK subfamily of serine/threonine protein kinases; positive regulators of canonical Wnt/b-catenin signaling.
WNK1	ENST00000537687	P140	SNV	Chr12: 862958	c.227A>G	p.E76G	Damaging	Possibly damaging	
WNK1	ENST00000537687	P129, P136, P142	SNV	Chr12: 977560	c.2668G>A	p.G890R	Tolerated	Probably damaging	
WNK1	ENST00000537687	P151	SNV	Chr12: 1005634	c.6761C>T	p.P2254L	Damaging	Probably damaging	

^1^Chromosomal positions are based on hg19 and dbSNP Build 137;

^2^Functional data are from PubMed searches and the 1000 Genomes Project.

**Fig 2:**
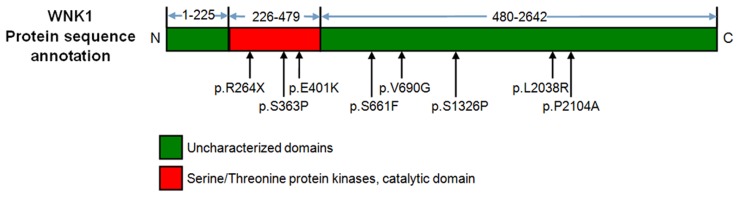
Location of 8 novel variants identified in 161 POP patients through Sanger sequencing. The p.E401K variant was detected in 2 unrelated cases.

*WNK1* contains 28 exons and encodes 2,642 amino acids, including one serine/threonine protein kinase catalytic domain across 254 residues (Fig. 1E). To determine whether POP patients carried other causative variants, we performed scanning of the entire coding regions of *WNK1* by Sanger sequencing in a total of 161 POP patients. A total of 8 novel variants and the c.2668G > A (p.G890R) variant identified by exome sequencing were found 11 patients (Fig. 2 and S4 Table). The eight novel variants were as follows: c.790A>T (p.R264X), c.1087T>C (p.S363P), c.1201G>A (p.E401K), c.1982C>T (p.S661F), c.2069T>G (p.V690G), c.3976T>C (p.S1326P), c.6113T>G (p.L2038R) and c.6310C>G (p.P2104A). Among them, c.1201G>A (p.E401K) was detected in 2 unrelated cases. Besides, the variant identified from exome sequencing, c.2668G > A (p.G890R), was detected in additional 2 patients. All these variants were absent in the 231 healthy controls, which suggested that they were not common polymorphisms but were specific to POP.

### Fibroblasts from POP patients showed irregular alignment

The pelvic viscera are primarily supported by the fibromuscular connective tissue of the female pelvic floor. Collagen and elastin, which are produced by fibroblasts, form the fibrillar ECM and provide perpendicular force to maintain regular fibroblast alignment [28,29]. One pathogenic mechanism of POP is reduced collagen synthesis and collagen cross-linking, which form the fragile support structures of the pelvic viscera.

**Fig 3:**
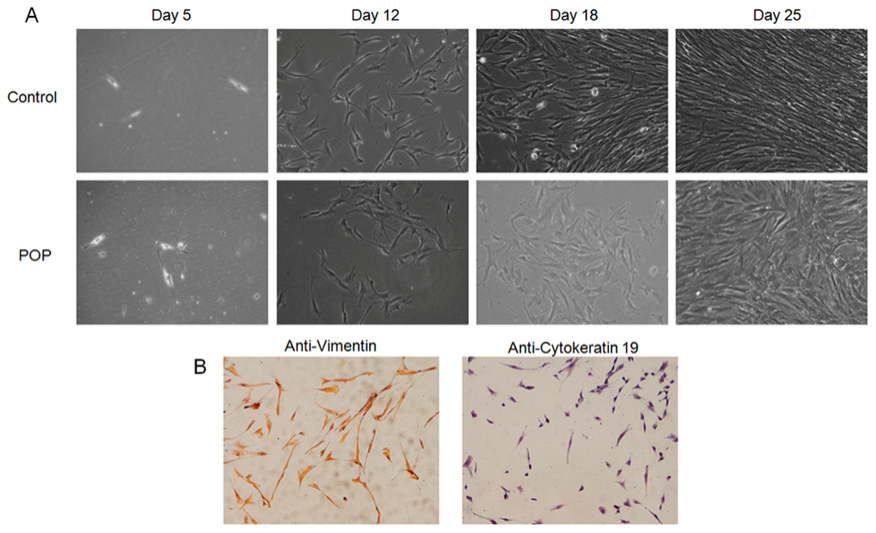
Fibroblasts from POP patients who carried the c.2668G > A (p.G890R) mutation of *WNK1* showing irregular alignment. (A) Fibroblasts from utero-sacral ligaments were cultured *in vitro* for 25 days. Control: healthy individuals; POP: POP cases with a *WNK1* mutation. (B) Most cells were vimentin positive and cytokeratin negative, which suggests they were fibroblasts.

We got utero-sacral ligament tissues from two POP patients with c.2668G > A (p.G890R) mutation who underwent surgical operation, and cultured fibroblasts *in vitro* for 25 days and determined that these cells showed loose and irregular alignment compared with fibroblasts from healthy women (Fig. 3A). To exclude the possibility that these cells were not fibroblasts but smooth muscle cells, which could have been brought into the culture because of incomplete adherence during cell separation, we performed IHC using anti-Cytokeratin and anti-Vimentin antibodies specific for fibroblasts and smooth muscle cells, respectively. The results showed that more than 90% of the cells were cytokeratin positive and vimentin negative, which suggests most of the cells were fibroblasts (Fig. 3B).

## Discussion

Understanding the pathogenetic mechanisms of a disease primarily depends on the identification of susceptibility variants that are correlated with the phenotype. Exome sequencing can sequence whole coding regions, which harbor approximately 85% of disease-causing mutations [30], with unparalleled specificity and accuracy; thus, it has greatly improved our understanding of the genetic pathology of diseases, including monogenic disorders and complex diseases. Reports of exome sequencing used to identify disease variants have increased exponentially [31–34]. This study was the first to identify genetic variations in POP patients using exome sequencing. The sequence data per individual was as high as 10.9 Gb, which provides sufficient sequencing depth to discover the susceptibility variants. In addition, our study searched for POP susceptibility genes across the whole exome, which provided more comprehensive variant information compared with candidate gene association studies; therefore, our results should also be more convincing.

In this study, two missense mutations of *WNK1*, c.2668G > A (p.G890R) and c.6761C> T (p.P2254L), were identified through exome sequencing and 8 additional variants were detected by Sanger sequencing. The *WNK1* gene is located on chromosome 12p13.3 and spans 156 Kb of genomic DNA [35]. WNK1 is a member of the serine/threonine protein kinase family that contains a small N-terminal domain followed by the kinase domain and a long C-terminal tail [36]. Mutations in *WNK1* have been discovered in a number of diseases, such as hypertension [37], pseudohypoaldosteronism type 2 (MIM 145260) [38], and hereditary sensory neuropathy type 2 [39]. In this study, we reported, for the first time, that rare mutations in *WNK1* could result in POP in a sample of the Chinese Han population.

WNK1 has been reported to regulate multiple intracellular signaling pathways. For example, WNK1 not only activated ERK5 through epidermal growth factor receptors[40] but also played an important role in G protein-coupled receptor signaling[41]. More importantly, WNK kinases activated canonical Wnt/b-catenin signaling [25], which was determined to be involved in the pathogenesis of POP [27].

Furthermore, we demonstrated that fibroblasts from POP patients who carried the c.2668G > A (p.G890R) mutation of WNK1 exhibited irregular alignment compared with healthy individuals, which suggests that a wild-type WNK1 might be essential to maintain the normal functions of fibroblasts. Fibroblasts were mechanosensitive, which could produce anabolic proteins, such as collagens. With the assistance of anabolic proteins, fibroblasts respond to mechanical stimuli by remodelling their actin cytoskeleton [28]. Alejandra et al. reported that the actin cytoskeleton of fibroblasts aligned perpendicular to external mechanical force, especially in the presence of collagen I [10], which then provided the main source of biological force to maintain the correct position of the uterus. If the fibroblast cell alignment become irregular which might be as a result of reduced collagen content in ECM, POP might occur [9,10]. Our result might explain one possible mechanism through which WNK1 dysfunction lead to POP onset.

However, we could only obtain fibroblasts with the c.2668G> A (p.G890R) mutation in our study; whether fibroblasts with the c.6761C> T (p.P2254L) mutation could cause similar phenomena in fibroblasts remains to be elucidated. It would be useful to systematically investigate the impacts on fibroblasts of different mutations of *WNK1*.

In summary, *WNK1*, a new POP susceptibility gene, may participate in a new pathogenic POP pathway. Our study provides evidence for prenatal genetic screening and the early diagnosis of POP, as well as a theoretical basis for clinical treatment and drug development.

### URL

1000 Genomes Project, http://www.1000genomes.org; dbSNP, http://www.ncbi.nlm.nih.gov/projects/SNP/; SIFT, http://sift.jcvi.org/; PolyPhen-2, http://genetics.bwh.harvard.edu/pph2/.

## Supporting Information

S1 Table Basic information of three specific primers targeting four variants of *WNK1*. (DOC)

S2 Table Twenty-eight PCR primers targeting the entire coding regions of *WNK1*. (DOC)

S3 Table Overview of candidate genes with variants in no less than two individuals. (DOC)

S4 Table Overview of 9 variants identified through Sanger sequencing in 161 unrelated POP patients. (DOC)
