# Supplementary material for: Exome Sequencing Identifies a Novel Gene, WNK1, for Susceptibility to Pelvic Organ Prolapse (POP)
Source: PLoS One. 2015 Mar 4;10(3):e0119482. doi: 10.1371/journal.pone.0119482 (PMC4349638; doi:10.1371/journal.pone.0119482)
Supplement: S1 Table — (DOC) [file pone.0119482.s001.doc]

**Table S1 Basic information of three specific primers targeting four variants of *WNK1***

| Primer No. | Variants covered | Oligonucleotide primers (5’ →3’) | Size of PCR product (bp) |
| --- | --- | --- | --- |
| Primer 1 | chr12: 862735  chr12: 862958 | F TGCTGAGTGAGGCGTCGT | 665 |
|  |  | R TTTGCTCCCCACAAGGCT |  |
| Primer 2 | chr12: 977560 | F AACTCACCACTTCCAACC | 811 |
|  |  | R TCCGATAGGCTCTACCAC |  |
| Primer 3 | chr12: 1005634 | F GTGCCCACAAAACTACTG | 306 |
|  |  | R CATCATCCACATCCCTAC |  |
