# Supplementary material for: Exome Sequencing Identifies a Novel Gene, WNK1, for Susceptibility to Pelvic Organ Prolapse (POP)
Source: PLoS One. 2015 Mar 4;10(3):e0119482. doi: 10.1371/journal.pone.0119482 (PMC4349638; doi:10.1371/journal.pone.0119482)
Supplement: S2 Table — (DOC) [file pone.0119482.s002.doc]

**Table S2 Twenty-eight PCR primers targeting the entire coding regions of *WNK1***

| Primer No. | Oligonucleotide primers (5’ →3’) | Size of PCR product (bp) |
| --- | --- | --- |
| Exon 1-1 | F TGCTGAGTGAGGCGTCGT | 665 |
|  | R TTTGCTCCCCACAAGGCT |  |
| Exon 1-2 | F ACCACTACCACCACTGAG | 738 |
|  | R TTATCCTATCCCAACACC |  |
| Exon 2 | F CCTCCAAAGCCTATGTTC | 565 |
|  | R GTGAAACTGGTAGACCTGAA |  |
| Exon 3 | F CAAAGCAACAAACTCCTA | 456 |
|  | R GATTCCAAGAAGCACAAA |  |
| Exon 4 | F CTCCCCCTTTCCTTTTTTC | 325 |
|  | R GCCCCAAGTTCAACAATCA |  |
| Exon 5 | F TATGGAAGTAAGTGACCC | 392 |
|  | R CCGTAGAATGTGTGTTGT |  |
| Exon 6 | F TAAACCTCTGTAGGCACCC | 486 |
|  | R CACGGCACTTCATCAACT |  |
| Exon 7 | F GCAGATTTTACAGTTCGGT | 515 |
|  | R TCCACTTCCCAAGATACG |  |
| Exon 8 | F TGCTGTTTCCCTTACTCC | 523 |
|  | R AGACTCATACTGTTACTTGGTTT |  |
| Exon 9 | F TACAGCCTGAGCAGCATT | 652 |
|  | R AGCCTGGGTGACAGAGTG |  |
| Exon 10 | F GTTGGGGTGAGGGAGATA | 751 |
|  | R GGAATGTCATAGGTTCAGTTA |  |
| Exon 11 | F TAGCCTCTTTCTCTCCTGC | 713 |
|  | R AAACTCTGTGGTGCCCTC |  |
| Exon 12 | F TTGGCTACTATTCTTCTTTG | 476 |
|  | R GCCTGTTTCTTTCTCCTT |  |
| Exon 13/14 | F CGTAGTGGGGAGGGATAA | 568 |
|  | R AGTGGTTTGGATTTGATGAC |  |
| Exon 15 | F CGATAACACTATGCTTCC | 390 |
|  | R CTAAATCACAGGTTGGGT |  |
| Exon 16/17 | F AATAAAACTATCACACCCAACC | 726 |
|  | R CTACAGAAGCCTGCCACC |  |
| Exon 18 | F CTGGGTGAGAGAATGAGA | 294 |
|  | R GCAGAGGCACTGTAGAAT |  |
| Exon 19-1 | F ACAATCTTTTGAATCCATCC | 807 |
|  | R CTAGTGAGTGTGCGCTAACT |  |
| Exon 19-2 | F GGGAGCCACATTAACATC | 1023 |
|  | R ATCCTGCCACACTTGAAA |  |
| Exon 20 | F CTCACGGACTTGATTTTC | 388 |
|  | R GTTTGCTTTGCTGTTTTCT |  |
| Exon 21 | F AAAACAGTGTGTCCTATCTAA | 454 |
|  | R CCTCCTCTGAAACAAAAAT |  |
| Exon 22 | F GGGATTACAGGTGTGAGC | 380 |
|  | R AATGTGTCTTTGAAACGGT |  |
| Exon 23 | F ATGGAGGGAGGAGATAAG | 302 |
|  | R CCCAAATAGGTAGGCAGTA |  |
| Exon 24 | F TAGTCAAAAGTGGAGGCG | 972 |
|  | R TGTGTGGTGGGGAGGTAT |  |
| Exon 25 | F GCAGATTGGGTCAGAGTA | 558 |
|  | R GACAGAAACAAAAGATAGGA |  |
| Exon 26 | F TCTATTGAGTCTTAGTGTCATTCC | 451 |
|  | R AGGCTTTTGTCCCAGGTAT |  |
| Exon 27 | F ACCAAAACAAATGGCTAAAC | 379 |
|  | R TGCGGCAAAAGAGAGACT |  |
| Exon 28 | F AAACAAGGCAAAAATCCC | 767 |
|  | R CCCCTCTGGCAAGAAATA |  |
