# Supplementary material for: Exome Sequencing Identifies a Novel Gene, WNK1, for Susceptibility to Pelvic Organ Prolapse (POP)
Source: PLoS One. 2015 Mar 4;10(3):e0119482. doi: 10.1371/journal.pone.0119482 (PMC4349638; doi:10.1371/journal.pone.0119482)
Supplement: S3 Table — (DOC) [file pone.0119482.s003.doc]

**Table S3** Overview of candidate genes with variants in no less than two individuals

| Gene Symbol | Transcription ID | Individuals sharing the variant | Mutation type | Chr. (position)^1^ | Nucleotide change | Protein level change | SIFT prediction | PolyPhen-2 prediction |
| --- | --- | --- | --- | --- | --- | --- | --- | --- |
| TTC40 | ENST00000368586 | P140 | SNV | Chr10: 134622392 | c.7681C>G | p.L2561V | Tolerated | -/- |
|  |  | P136 | SNV | Chr10: 134736029 | c.1440G>T | p.Q480H | Damaging | Probably damaging |
| ATG2A | ENST00000421419 | P51 | SNV | Chr11: 64662809 | c.5539G>A | p.A1847T | Damaging | Probably damaging |
|  |  | P140 | SNV | Chr11: 64662889 | c.5459T>A | p.I1820N | Damaging | Possibly damaging |
|  |  | P28 | SNV | Chr11: 64670052 | c.3713T>C | p.M1238T | Tolerated | Benign |
| KRT81 | ENST00000327741 | P51 | SNV | Chr12: 52680123 | c.1434C>A | p.C478X | Tolerated | Possibly damaging |
|  |  | P142 | SNV | Chr12: 52680198 | c.1359C>A | p.C453X | Tolerated | Benign |
|  |  | P153 | SNV | Chr12: 52681438 | c.968A>G | p.N323S | Tolerated | Benign |
| CABP1 | ENST00000453000 | P28, P136, P153 | Indel | Chr12: 121093654 | c.41_42del | frameshift | -/- | -/- |
| GALNT9 | ENST00000535228 | P28, P129 | Indel | Chr12: 132811499 | c.188del | frameshift | -/- | -/- |
| WNK1 | ENST00000537687 | P153 | SNV | Chr12: 862735 | c.4T>A | p.S2T | Damaging | Probably damaging |
|  |  | P140 | SNV | Chr12: 862958 | c.227A>G | p.E76G | Damaging | Possibly damaging |
|  |  | P129, P136, P142 | SNV | Chr12: 977560 | c.2668G>A | p.G890R | Tolerated | Probably damaging |
|  |  | P151 | SNV | Chr12: 1005634 | c.6761C>T | p.P2254L | Damaging | Probably damaging |
| DACH1 | ENST00000359684 | P28, P151 | Indel | Chr13: 72440660 | c.247_248insAGG | p.82G_83SinsG | -/- | -/- |
| IRF2BPL | ENST00000238647 | P28, P51, P142,P151 | Indel | Chr14: 77493792 | c.340_342del | p.Q144del | -/- | -/- |
| KBTBD13 | ENST00000432196 | P28 | SNV | Chr15: 65369381 | c.228C>G | p.D76E | Tolerated | Benign |
|  |  | P140 | SNV | Chr15: 65369512 | c.359A>T | p.D120V | Damaging | Probably damaging |
|  |  | P151 | SNV | Chr15: 65369558 | c.405G>T | p.E135D | Damaging | Possibly damaging |
| CSPG4 | ENST00000308508 | P28, P140 | SNV | Chr15: 75982324 | c.1082A>G | p.N361S | Damaging | Possibly damaging |

^1^Chromosomal positions are based on hg19 and dbSNP Build 137.
