# Supplementary material for: Exome Sequencing Identifies a Novel Gene, WNK1, for Susceptibility to Pelvic Organ Prolapse (POP)
Source: PLoS One. 2015 Mar 4;10(3):e0119482. doi: 10.1371/journal.pone.0119482 (PMC4349638; doi:10.1371/journal.pone.0119482)
Supplement: S4 Table — (DOC) [file pone.0119482.s004.doc]

**Table S4** **Overview of 9 variants identified through Sanger sequencing in 161 unrelated POP patients**

| Chr. (position)^1^ | CDS locus | Codon change | Amino acid change | Subject ID (case) |
| --- | --- | --- | --- | --- |
| Chr12: 922838 | c.790A>T | AGA>tGA | p.R264X | P31 |
| Chr12: 936362 | c.1087T>C | TCA>cCA | p.S363P | P147 |
| Chr12: 939215 | c.1201G>A | GAA>aAA | p.E401K | P72, P119 |
| Chr12: 971279 | c.1982C>T | TCC>TtC | p.S661F | P63 |
| Chr12: 971366 | c.2069T>G | GTC>GgC | p.V690G | P104 |
| Chr12: 977560 | c.2668G>A | GGG>aGG | p.G890R^2^ | P19, P101 |
| Chr12: 990942 | c.3976T>C | TCA>cCA | p.S1326P | P72 |
| Chr12: 996442 | c.6113T>G | CTG>CgG | p.L2038R | P93 |
| Chr12: 999703 | c.6310C>G | CCT>gCT | p.P2104A | P18 |

^1^Chromosomal positions are based on hg19 and dbSNP Build 137

^2^ This variant was first identified through exome sequencing
